# Diethyl 7,8,18,19-tetra­methyl-2,13-dioxo­hexa­cyclo­[10.10.2.0^3,24^.0^5,10^.0^14,23^.0^16,21^]tetra­cosa-5,7,9,16,18,20-hexa­ene-23,24-dicarboxyl­ate

**DOI:** 10.1107/S1600536810022038

**Published:** 2010-06-16

**Authors:** Jungang Wang, Jiacheng Xiang, Liping Cao

**Affiliations:** a Key Laboratory of Pesticides and Chemical Biology of Ministry of Education, College of Chemistry, Central China Normal University, Wuhan 430079, People’s Republic of China

## Abstract

The asymmetric unit of the title compound, C_30_H_34_N_4_O_6_, contains two independent mol­ecules. In one independent mol­ecule, the two eth­oxy­carbonyl groups are each disordered over two conformations with occupancy ratios of 0.586 (2):0.414 (2) and 0.508 (2):0.492 (2). The crystal packing exhibits weak inter­molecular C—H⋯O hydrogen bonds.

## Related literature

For the preparation and the crystal engineering studies on the title compound, see: Wang *et al.* (2006 ▶). For glycoluril and its derivatives, see: Freeman *et al.* (1981 ▶); Rebek (2005 ▶); Rowan *et al.* (1999 ▶); Wu *et al.* (2002 ▶).
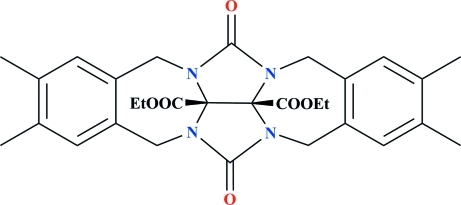


## Experimental

### 

#### Crystal data

C_30_H_34_N_4_O_6_
*M* *_r_* = 546.61
Monoclinic, 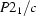

*a* = 23.4988 (12) Å
*b* = 11.6005 (6) Å
*c* = 21.2685 (11) Å
β = 107.145 (1)°
*V* = 5540.1 (5) Å^3^
*Z* = 8
Mo *K*α radiation
μ = 0.09 mm^−1^
*T* = 298 K
0.16 × 0.12 × 0.10 mm

#### Data collection

Bruker SMART 4K CCD area-detector diffractometer
67299 measured reflections
13767 independent reflections
6896 reflections with *I* > 2σ(*I*)
*R*_int_ = 0.052

#### Refinement

*R*[*F* ^2^ > 2σ(*F* ^2^)] = 0.047
*wR*(*F* ^2^) = 0.116
*S* = 0.86
13767 reflections
809 parameters
20 restraints
H-atom parameters constrained
Δρ_max_ = 0.17 e Å^−3^
Δρ_min_ = −0.18 e Å^−3^

### 

Data collection: *SMART* (Bruker, 1997 ▶); cell refinement: *SAINT* (Bruker, 1999 ▶); data reduction: *SAINT*; program(s) used to solve structure: *SHELXS97* (Sheldrick, 2008 ▶); program(s) used to refine structure: *SHELXL97* (Sheldrick, 2008 ▶); molecular graphics: *SHELXTL* (Sheldrick, 2008 ▶); software used to prepare material for publication: *SHELXTL*.

## Supplementary Material

Crystal structure: contains datablocks I, global. DOI: 10.1107/S1600536810022038/cv2719sup1.cif

Structure factors: contains datablocks I. DOI: 10.1107/S1600536810022038/cv2719Isup2.hkl

Additional supplementary materials:  crystallographic information; 3D view; checkCIF report

## Figures and Tables

**Table 1:** Hydrogen-bond geometry (Å, °)

*D*—H⋯*A*	*D*—H	H⋯*A*	*D*⋯*A*	*D*—H⋯*A*
C9*A*—H9*A*1⋯O2*B*^i^	0.97	2.31	3.205 (2)	153
C22*B*—H22*A*⋯O1*A*^ii^	0.97	2.41	3.375 (2)	178
C29*B*—H29*A*⋯O1*B*^iii^	0.96	2.53	3.441 (3)	160

Symmetry codes: (i) 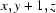; (ii) 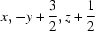; (iii) 
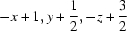
.

**Fig. 1.**
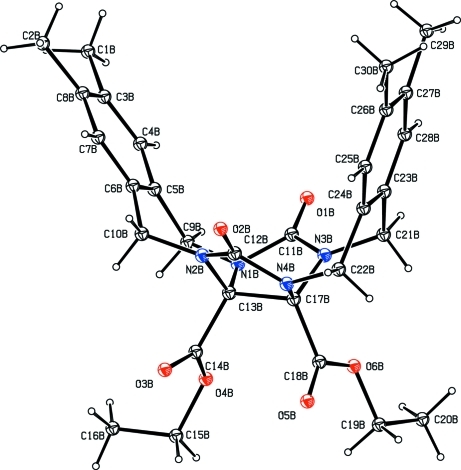
A view of (I), showing one independent molecule with the atom-labelling scheme and 30% probability displacement ellipsoids.

## supplementary crystallographic information

### Comment

Glycoluril and its derivatives are widely used as building blocks for studies of self-assembly in homogeneous solution (Freeman *et al.*, 1981; Rebek, 2005; Rowan *et al.*, 1999; Wu *et al.*, 2002). As a part of our ongoing investigation into glycoluril derivatives (Wang *et al.*, 2006), here we report the structure of the title compound (I) (Fig. 1).

The asymmetric unit of (I) contains two independent molecules. In one independent molecule, two ethoxy carbonyl groups are disordered over two conformations each with ratios 0.586 (2)/0.414 (2) and 0.508 (2)/0.492 (2), respectively. The crystal packing exhibits weak intermolecular C—H···O hydrogen bonds (Table 1).

### Experimental

The title compound was synthesized according to the reported method (Wang *et al.*; 2006). Crystals of (I) suitable for X-ray diffraction were grown by slow evaporation of a dichloromethane-methanol (4:1) solution of the title compound under 293 K.

### Refinement

All H-atoms were positioned geometrically and constrained to ride on their parent atoms, with d(C—H) = 0.97 Å, Uĩso~ = 1.2U~eq~ (C) for CH~2~ and d(C—H) = 0.96 Å, Uĩso~ = 1.5U~eq~ (C) for CH~3~ atoms. In one independent molecule, two ethoxy carbonyl groups were treated as disordered over two conformations. The occupancies of the disordered positions C15A/ C15', C16A/ C16', O3A/O3' and O4A/O4' were refined to 0.508 (2) / 0.492 (2), while those for C19A/ C19', C20A/ C20', O5A/O5' and O6A/O6' were refined to 0.414 (2) / 0.586 (2).

### Crystal data

**Table:**

C_30_H_34_N_4_O_6_	*F*(000) = 2320
*M**_r_* = 546.61	*D*_x_ = 1.311 Mg m^−^^3^
Monoclinic, *P*2_1_/*c*	Mo *K*α radiation, λ = 0.71073 Å
Hall symbol: -P 2ybc	Cell parameters from 8852 reflections
*a* = 23.4988 (12) Å	θ = 2.3–22.3°
*b* = 11.6005 (6) Å	µ = 0.09 mm^−^^1^
*c* = 21.2685 (11) Å	*T* = 298 K
β = 107.145 (1)°	Block, colourless
*V* = 5540.1 (5) Å^3^	0.16 × 0.12 × 0.10 mm
*Z* = 8	

### Data collection

**Table:**

Bruker SMART 4K CCD area-detector diffractometer	6896 reflections with *I* > 2σ(*I*)
Radiation source: fine-focus sealed tube	*R*_int_ = 0.052
graphite	θ_max_ = 28.3°, θ_min_ = 1.8°
phi and ω scans	*h* = −31→31
67299 measured reflections	*k* = −15→15
13767 independent reflections	*l* = −28→28

### Refinement

**Table:**

Refinement on *F*^2^	Primary atom site location: structure-invariant direct methods
Least-squares matrix: full	Secondary atom site location: difference Fourier map
*R*[*F*^2^ > 2σ(*F*^2^)] = 0.047	Hydrogen site location: inferred from neighbouring sites
*wR*(*F*^2^) = 0.116	H-atom parameters constrained
*S* = 0.86	*w* = 1/[σ^2^(*F*_o_^2^) + (0.0539*P*)^2^] where *P* = (*F*_o_^2^ + 2*F*_c_^2^)/3
13767 reflections	(Δ/σ)_max_ = 0.002
809 parameters	Δρ_max_ = 0.17 e Å^−^^3^
20 restraints	Δρ_min_ = −0.18 e Å^−^^3^

### Special details

**Table:**

Geometry. All e.s.d.'s (except the e.s.d. in the dihedral angle between two l.s. planes) are estimated using the full covariance matrix. The cell e.s.d.'s are taken into account individually in the estimation of e.s.d.'s in distances, angles and torsion angles; correlations between e.s.d.'s in cell parameters are only used when they are defined by crystal symmetry. An approximate (isotropic) treatment of cell e.s.d.'s is used for estimating e.s.d.'s involving l.s. planes.
Refinement. Refinement of *F*^2^ against ALL reflections. The weighted *R*-factor *wR* and goodness of fit *S* are based on *F*^2^, conventional *R*-factors *R* are based on *F*, with *F* set to zero for negative *F*^2^. The threshold expression of *F*^2^ > σ(*F*^2^) is used only for calculating *R*-factors(gt) *etc*. and is not relevant to the choice of reflections for refinement. *R*-factors based on *F*^2^ are statistically about twice as large as those based on *F*, and *R*- factors based on ALL data will be even larger.

### Fractional atomic coordinates and isotropic or equivalent isotropic displacement parameters (Å^2^)

**Table:**

	*x*	*y*	*z*	*U*_iso_*/*U*_eq_	Occ. (<1)
O1A	0.18810 (5)	1.18390 (11)	0.17156 (6)	0.0609 (3)	
O2A	0.13366 (5)	0.74461 (11)	0.24984 (6)	0.0633 (4)	
N1A	0.18396 (6)	1.08832 (11)	0.26470 (7)	0.0445 (3)	
N2A	0.15077 (6)	0.91667 (12)	0.30754 (7)	0.0454 (3)	
N3A	0.10001 (6)	1.11326 (12)	0.18400 (7)	0.0479 (4)	
N4A	0.08583 (6)	0.91354 (12)	0.20740 (7)	0.0494 (4)	
C1A	0.42409 (8)	0.89433 (17)	0.27568 (10)	0.0669 (6)	
H1A1	0.4500	0.8318	0.2953	0.100*	
H1A2	0.4435	0.9662	0.2910	0.100*	
H1A3	0.4150	0.8903	0.2287	0.100*	
C2A	0.38198 (9)	0.67344 (17)	0.32156 (10)	0.0693 (6)	
H2A1	0.3824	0.6491	0.2785	0.104*	
H2A2	0.3633	0.6153	0.3408	0.104*	
H2A3	0.4221	0.6851	0.3488	0.104*	
C3A	0.36724 (7)	0.88602 (16)	0.29477 (8)	0.0489 (4)	
C4A	0.33249 (7)	0.98434 (15)	0.29049 (8)	0.0483 (4)	
H4A	0.3451	1.0521	0.2753	0.058*	
C5A	0.28011 (7)	0.98636 (14)	0.30767 (8)	0.0423 (4)	
C6A	0.26109 (7)	0.88475 (15)	0.33059 (8)	0.0439 (4)	
C7A	0.29503 (7)	0.78633 (15)	0.33343 (8)	0.0483 (4)	
H7A	0.2820	0.7181	0.3476	0.058*	
C8A	0.34788 (7)	0.78424 (15)	0.31616 (8)	0.0486 (4)	
C9A	0.24609 (7)	1.09810 (14)	0.30318 (8)	0.0479 (4)	
H9A1	0.2482	1.1235	0.3473	0.058*	
H9A2	0.2650	1.1565	0.2835	0.058*	
C10A	0.20665 (7)	0.88039 (16)	0.35484 (9)	0.0528 (5)	
H10C	0.2019	0.8020	0.3683	0.063*	
H10D	0.2139	0.9289	0.3936	0.063*	
C11A	0.16072 (8)	1.13303 (14)	0.20341 (9)	0.0459 (4)	
C12A	0.12450 (7)	0.84661 (17)	0.25404 (9)	0.0482 (4)	
C13A	0.13984 (7)	1.03574 (15)	0.29051 (8)	0.0451 (4)	
C14A	0.13113 (9)	1.1126 (2)	0.34619 (10)	0.0588 (5)	
C15A	0.1006 (6)	1.1414 (7)	0.4389 (5)	0.081 (3)	0.508 (14)
H15C	0.0701	1.1979	0.4195	0.098*	0.508 (14)
H15D	0.1374	1.1816	0.4604	0.098*	0.508 (14)
C16A	0.0821 (7)	1.0681 (10)	0.4872 (5)	0.107 (3)	0.508 (14)
H16G	0.0433	1.0367	0.4667	0.160*	0.508 (14)
H16H	0.0811	1.1141	0.5243	0.160*	0.508 (14)
H16I	0.1101	1.0064	0.5016	0.160*	0.508 (14)
O3A	0.1475 (4)	1.2123 (5)	0.3507 (6)	0.077 (2)	0.508 (14)
O4A	0.1090 (5)	1.0641 (9)	0.3884 (5)	0.067 (2)	0.508 (14)
C16'	0.0627 (3)	1.1133 (12)	0.4607 (7)	0.101 (4)	0.492 (14)
H16F	0.0413	1.1644	0.4264	0.151*	0.492 (14)
H16E	0.0634	1.1449	0.5027	0.151*	0.492 (14)
H16D	0.0432	1.0395	0.4552	0.151*	0.492 (14)
C15'	0.1248 (3)	1.0992 (11)	0.4576 (4)	0.074 (2)	0.492 (14)
H15E	0.1438	1.1742	0.4617	0.088*	0.492 (14)
H15F	0.1471	1.0520	0.4943	0.088*	0.492 (14)
O3'	0.1234 (6)	1.2148 (7)	0.3406 (6)	0.107 (3)	0.492 (14)
O4'	0.1264 (5)	1.0456 (9)	0.3960 (4)	0.063 (2)	0.492 (14)
C17A	0.08463 (7)	1.03234 (15)	0.22728 (8)	0.0476 (4)	
C18A	0.02543 (9)	1.0647 (2)	0.23998 (11)	0.0618 (5)	
C19A	−0.0582 (5)	0.9725 (15)	0.2662 (6)	0.076 (3)	0.414 (14)
H19D	−0.0841	0.9105	0.2440	0.091*	0.414 (14)
H19C	−0.0782	1.0451	0.2514	0.091*	0.414 (14)
C20A	−0.0465 (6)	0.961 (2)	0.3392 (5)	0.125 (6)	0.414 (14)
H20A	−0.0268	0.8892	0.3537	0.187*	0.414 (14)
H20B	−0.0836	0.9631	0.3495	0.187*	0.414 (14)
H20C	−0.0217	1.0236	0.3610	0.187*	0.414 (14)
O5A	0.0137 (4)	1.1627 (7)	0.2523 (6)	0.083 (2)	0.414 (14)
O6A	−0.0033 (5)	0.9682 (9)	0.2497 (6)	0.076 (3)	0.414 (14)
C19'	−0.0466 (4)	1.0092 (8)	0.2890 (5)	0.072 (2)	0.586 (14)
H19E	−0.0799	1.0184	0.2496	0.087*	0.586 (14)
H19F	−0.0427	1.0790	0.3151	0.087*	0.586 (14)
C20'	−0.0562 (4)	0.9078 (7)	0.3277 (5)	0.084 (2)	0.586 (14)
H20D	−0.0629	0.8403	0.3003	0.126*	0.586 (14)
H20E	−0.0903	0.9213	0.3428	0.126*	0.586 (14)
H20F	−0.0217	0.8964	0.3648	0.126*	0.586 (14)
O5'	−0.0028 (4)	1.1489 (7)	0.2157 (5)	0.108 (2)	0.586 (14)
O6'	0.0079 (3)	0.9872 (6)	0.2718 (4)	0.0645 (18)	0.586 (14)
C21A	0.06619 (8)	1.11686 (16)	0.11404 (9)	0.0600 (5)	
H21C	0.0758	1.1880	0.0955	0.072*	
H21D	0.0241	1.1192	0.1105	0.072*	
C22A	0.04665 (8)	0.87020 (17)	0.14544 (9)	0.0606 (5)	
H22C	0.0068	0.8994	0.1397	0.073*	
H22D	0.0450	0.7868	0.1477	0.073*	
C23A	0.07681 (8)	1.01776 (17)	0.07294 (9)	0.0543 (5)	
C24A	0.06599 (7)	0.90349 (17)	0.08606 (9)	0.0542 (5)	
C25A	0.07319 (8)	0.81895 (18)	0.04264 (10)	0.0629 (5)	
H25A	0.0651	0.7428	0.0507	0.075*	
C26A	0.09188 (8)	0.8428 (2)	−0.01213 (10)	0.0657 (6)	
C27A	0.10430 (9)	0.9561 (2)	−0.02375 (10)	0.0689 (6)	
C28A	0.09629 (8)	1.04109 (19)	0.01882 (9)	0.0661 (5)	
H28A	0.1044	1.1172	0.0106	0.079*	
C29A	0.12856 (11)	0.9883 (3)	−0.08023 (11)	0.1068 (9)	
H29D	0.1298	1.0707	−0.0838	0.160*	
H29E	0.1032	0.9569	−0.1205	0.160*	
H29F	0.1680	0.9576	−0.0721	0.160*	
C30A	0.09864 (10)	0.7452 (2)	−0.05647 (11)	0.0939 (8)	
H30D	0.0744	0.7602	−0.1006	0.141*	
H30F	0.0862	0.6743	−0.0413	0.141*	
H30E	0.1396	0.7391	−0.0557	0.141*	
O1B	0.43937 (5)	0.04574 (12)	0.71998 (6)	0.0632 (4)	
O2B	0.28886 (5)	0.12011 (11)	0.46037 (6)	0.0611 (3)	
O3B	0.23181 (6)	−0.17082 (13)	0.54130 (7)	0.0830 (5)	
O4B	0.27176 (5)	−0.20261 (11)	0.64928 (6)	0.0589 (3)	
O5B	0.18946 (6)	0.03919 (13)	0.62346 (7)	0.0790 (4)	
O6B	0.26075 (5)	0.05723 (12)	0.71918 (6)	0.0662 (4)	
N1B	0.36696 (6)	−0.07053 (12)	0.65219 (6)	0.0456 (3)	
N2B	0.32046 (6)	−0.02124 (13)	0.53878 (6)	0.0471 (4)	
N3B	0.34505 (6)	0.11166 (13)	0.66635 (6)	0.0463 (4)	
N4B	0.26966 (6)	0.12751 (12)	0.56039 (6)	0.0454 (3)	
C1B	0.59664 (8)	−0.2226 (2)	0.59397 (11)	0.0791 (6)	
H1B1	0.6071	−0.2400	0.6401	0.119*	
H1B2	0.6215	−0.1614	0.5868	0.119*	
H1B3	0.6023	−0.2899	0.5702	0.119*	
C2B	0.53769 (9)	−0.16490 (19)	0.45324 (10)	0.0748 (6)	
H2B1	0.5753	−0.1265	0.4699	0.112*	
H2B2	0.5147	−0.1274	0.4137	0.112*	
H2B3	0.5442	−0.2439	0.4438	0.112*	
C3B	0.53237 (8)	−0.18567 (15)	0.57005 (10)	0.0544 (5)	
C4B	0.49900 (8)	−0.17902 (14)	0.61356 (9)	0.0514 (4)	
H4B	0.5180	−0.1931	0.6578	0.062*	
C5B	0.43871 (7)	−0.15249 (14)	0.59491 (8)	0.0460 (4)	
C6B	0.41070 (8)	−0.12653 (14)	0.52904 (9)	0.0478 (4)	
C7B	0.44481 (8)	−0.12908 (14)	0.48547 (9)	0.0530 (5)	
H7B	0.4267	−0.1091	0.4418	0.064*	
C8B	0.50443 (8)	−0.15993 (15)	0.50415 (10)	0.0535 (5)	
C9B	0.40622 (8)	−0.16434 (16)	0.64638 (9)	0.0536 (5)	
H9B1	0.3828	−0.2345	0.6372	0.064*	
H9B2	0.4358	−0.1743	0.6888	0.064*	
C10B	0.34481 (8)	−0.10271 (16)	0.50137 (8)	0.0548 (5)	
H10A	0.3372	−0.0734	0.4569	0.066*	
H10B	0.3235	−0.1751	0.4986	0.066*	
C11B	0.38948 (8)	0.03153 (16)	0.68311 (8)	0.0466 (4)	
C12B	0.29294 (7)	0.07911 (16)	0.51412 (8)	0.0458 (4)	
C13B	0.31232 (7)	−0.05049 (15)	0.60153 (8)	0.0450 (4)	
C14B	0.26692 (8)	−0.14919 (16)	0.59342 (10)	0.0539 (5)	
C15B	0.23022 (9)	−0.2983 (2)	0.64711 (10)	0.0754 (6)	
H15A	0.1907	−0.2761	0.6203	0.091*	
H15B	0.2282	−0.3137	0.6912	0.091*	
C16B	0.24875 (11)	−0.4051 (2)	0.61965 (12)	0.0959 (8)	
H16A	0.2461	−0.3930	0.5742	0.144*	
H16B	0.2230	−0.4675	0.6232	0.144*	
H16C	0.2891	−0.4237	0.6438	0.144*	
C17B	0.29055 (7)	0.06692 (15)	0.62221 (8)	0.0451 (4)	
C18B	0.24006 (9)	0.05387 (16)	0.65443 (10)	0.0536 (5)	
C19B	0.21676 (9)	0.0420 (2)	0.75488 (11)	0.0920 (8)	
H19A	0.1838	0.0950	0.7381	0.110*	
H19B	0.2013	−0.0360	0.7490	0.110*	
C20B	0.24566 (12)	0.0646 (2)	0.82460 (11)	0.0992 (8)	
H20G	0.2768	0.0093	0.8414	0.149*	
H20H	0.2169	0.0585	0.8484	0.149*	
H20I	0.2623	0.1409	0.8298	0.149*	
C21B	0.35170 (8)	0.23038 (15)	0.68850 (8)	0.0552 (5)	
H21A	0.3859	0.2363	0.7273	0.066*	
H21B	0.3168	0.2530	0.7009	0.066*	
C22B	0.25941 (7)	0.25230 (15)	0.55949 (9)	0.0539 (5)	
H22A	0.2378	0.2704	0.5907	0.065*	
H22B	0.2345	0.2742	0.5161	0.065*	
C23B	0.35967 (8)	0.31235 (15)	0.63671 (8)	0.0486 (4)	
C24B	0.31593 (8)	0.32328 (15)	0.57615 (8)	0.0488 (4)	
C25B	0.32564 (9)	0.39956 (16)	0.53058 (9)	0.0572 (5)	
H25B	0.2965	0.4073	0.4903	0.069*	
C26B	0.37723 (9)	0.46536 (16)	0.54247 (10)	0.0595 (5)	
C27B	0.42118 (8)	0.45323 (15)	0.60225 (10)	0.0554 (5)	
C28B	0.41137 (8)	0.37727 (15)	0.64819 (9)	0.0529 (5)	
H28B	0.4406	0.3694	0.6884	0.063*	
C29B	0.47928 (9)	0.51749 (17)	0.61727 (11)	0.0770 (6)	
H29A	0.5019	0.5041	0.6622	0.116*	
H29B	0.4716	0.5985	0.6105	0.116*	
H29C	0.5014	0.4909	0.5887	0.116*	
C30B	0.38487 (11)	0.5469 (2)	0.48971 (11)	0.0910 (7)	
H30A	0.4128	0.5147	0.4695	0.136*	
H30B	0.3994	0.6198	0.5093	0.136*	
H30C	0.3472	0.5578	0.4570	0.136*	

### Atomic displacement parameters (Å^2^)

**Table:**

	*U*^11^	*U*^22^	*U*^33^	*U*^12^	*U*^13^	*U*^23^
O1A	0.0681 (8)	0.0636 (8)	0.0582 (8)	−0.0060 (7)	0.0300 (7)	0.0106 (7)
O2A	0.0638 (8)	0.0445 (8)	0.0857 (10)	−0.0010 (7)	0.0284 (7)	0.0005 (7)
N1A	0.0429 (8)	0.0496 (9)	0.0438 (8)	−0.0020 (7)	0.0170 (7)	0.0057 (7)
N2A	0.0436 (8)	0.0477 (9)	0.0480 (9)	0.0007 (7)	0.0182 (7)	0.0055 (7)
N3A	0.0464 (9)	0.0530 (9)	0.0445 (9)	0.0031 (7)	0.0137 (7)	0.0044 (7)
N4A	0.0472 (9)	0.0493 (9)	0.0520 (9)	−0.0025 (7)	0.0151 (7)	−0.0024 (7)
C1A	0.0556 (12)	0.0763 (14)	0.0759 (14)	0.0014 (10)	0.0303 (11)	0.0031 (11)
C2A	0.0734 (14)	0.0653 (14)	0.0774 (14)	0.0149 (11)	0.0349 (12)	0.0068 (11)
C3A	0.0444 (10)	0.0584 (12)	0.0435 (10)	−0.0022 (9)	0.0125 (8)	−0.0014 (9)
C4A	0.0465 (10)	0.0502 (11)	0.0491 (11)	−0.0080 (9)	0.0154 (9)	0.0007 (8)
C5A	0.0428 (10)	0.0462 (10)	0.0376 (9)	−0.0026 (8)	0.0113 (8)	−0.0022 (8)
C6A	0.0431 (10)	0.0512 (11)	0.0376 (9)	0.0001 (8)	0.0123 (8)	0.0022 (8)
C7A	0.0536 (11)	0.0471 (11)	0.0445 (10)	−0.0025 (9)	0.0150 (9)	0.0054 (8)
C8A	0.0493 (11)	0.0541 (12)	0.0429 (10)	0.0053 (9)	0.0146 (8)	0.0014 (8)
C9A	0.0463 (10)	0.0500 (11)	0.0476 (10)	−0.0056 (8)	0.0141 (8)	−0.0017 (8)
C10A	0.0523 (11)	0.0596 (12)	0.0500 (11)	0.0023 (9)	0.0207 (9)	0.0126 (9)
C11A	0.0516 (11)	0.0429 (10)	0.0481 (11)	0.0023 (8)	0.0226 (9)	−0.0012 (8)
C12A	0.0424 (10)	0.0516 (12)	0.0590 (12)	−0.0040 (9)	0.0278 (9)	0.0033 (10)
C13A	0.0453 (10)	0.0485 (11)	0.0453 (10)	0.0009 (8)	0.0195 (8)	0.0017 (8)
C14A	0.0588 (13)	0.0670 (15)	0.0558 (13)	0.0052 (13)	0.0251 (10)	−0.0033 (12)
C15A	0.098 (7)	0.091 (6)	0.070 (5)	−0.001 (4)	0.046 (5)	−0.029 (4)
C16A	0.122 (9)	0.148 (9)	0.064 (5)	0.013 (5)	0.049 (6)	−0.004 (4)
O3A	0.088 (4)	0.048 (3)	0.109 (5)	−0.001 (2)	0.053 (4)	−0.018 (3)
O4A	0.074 (6)	0.074 (3)	0.068 (3)	−0.010 (3)	0.044 (4)	−0.015 (2)
C16'	0.090 (5)	0.125 (9)	0.099 (8)	0.006 (5)	0.047 (5)	−0.036 (6)
C15'	0.075 (5)	0.091 (7)	0.060 (4)	−0.009 (3)	0.027 (4)	−0.014 (3)
O3'	0.173 (9)	0.084 (4)	0.080 (4)	0.052 (4)	0.060 (6)	0.002 (3)
O4'	0.066 (5)	0.087 (4)	0.046 (3)	−0.005 (3)	0.034 (3)	−0.012 (2)
C17A	0.0428 (10)	0.0525 (11)	0.0505 (11)	0.0024 (8)	0.0184 (9)	0.0008 (9)
C18A	0.0478 (12)	0.0689 (15)	0.0725 (15)	0.0086 (11)	0.0235 (11)	0.0072 (13)
C19A	0.048 (5)	0.108 (9)	0.085 (7)	0.003 (4)	0.038 (5)	0.008 (5)
C20A	0.065 (5)	0.23 (2)	0.081 (6)	−0.023 (10)	0.028 (5)	0.033 (9)
O5A	0.071 (4)	0.070 (3)	0.122 (6)	0.018 (2)	0.050 (4)	−0.003 (4)
O6A	0.056 (4)	0.098 (5)	0.086 (6)	−0.005 (3)	0.038 (4)	−0.004 (3)
C19'	0.052 (4)	0.094 (5)	0.082 (6)	0.012 (3)	0.038 (4)	0.008 (4)
C20'	0.060 (5)	0.086 (4)	0.115 (6)	−0.003 (3)	0.041 (4)	0.025 (3)
O5'	0.089 (4)	0.109 (4)	0.150 (6)	0.050 (3)	0.071 (4)	0.057 (4)
O6'	0.046 (3)	0.070 (3)	0.088 (4)	0.0134 (19)	0.036 (3)	0.025 (3)
C21A	0.0614 (12)	0.0628 (13)	0.0524 (12)	0.0090 (10)	0.0114 (10)	0.0075 (10)
C22A	0.0513 (11)	0.0633 (13)	0.0652 (13)	−0.0107 (9)	0.0143 (10)	−0.0087 (10)
C23A	0.0453 (10)	0.0653 (13)	0.0478 (11)	0.0060 (9)	0.0068 (9)	0.0010 (10)
C24A	0.0407 (10)	0.0660 (14)	0.0523 (12)	−0.0004 (9)	0.0080 (9)	−0.0042 (10)
C25A	0.0504 (12)	0.0681 (14)	0.0630 (13)	0.0014 (10)	0.0058 (10)	−0.0080 (11)
C26A	0.0478 (11)	0.0940 (17)	0.0493 (12)	0.0139 (11)	0.0050 (10)	−0.0123 (12)
C27A	0.0582 (13)	0.0947 (18)	0.0524 (13)	0.0076 (12)	0.0142 (10)	−0.0006 (12)
C28A	0.0643 (13)	0.0793 (15)	0.0513 (12)	0.0026 (11)	0.0117 (10)	0.0072 (11)
C29A	0.112 (2)	0.151 (3)	0.0690 (16)	0.0029 (18)	0.0449 (15)	−0.0001 (16)
C30A	0.0838 (16)	0.117 (2)	0.0767 (16)	0.0254 (15)	0.0166 (13)	−0.0248 (14)
O1B	0.0422 (7)	0.0888 (10)	0.0525 (8)	−0.0113 (7)	0.0046 (6)	0.0012 (7)
O2B	0.0693 (8)	0.0764 (9)	0.0379 (7)	0.0098 (7)	0.0162 (6)	0.0073 (6)
O3B	0.0718 (9)	0.0992 (12)	0.0616 (9)	−0.0304 (8)	−0.0060 (8)	0.0069 (8)
O4B	0.0554 (8)	0.0702 (9)	0.0515 (8)	−0.0185 (6)	0.0164 (6)	0.0035 (7)
O5B	0.0440 (8)	0.1239 (13)	0.0725 (10)	−0.0137 (8)	0.0222 (7)	−0.0035 (9)
O6B	0.0534 (8)	0.1029 (11)	0.0513 (8)	0.0017 (7)	0.0292 (7)	0.0122 (7)
N1B	0.0379 (8)	0.0579 (9)	0.0410 (8)	−0.0008 (7)	0.0115 (7)	0.0014 (7)
N2B	0.0475 (8)	0.0596 (10)	0.0356 (8)	0.0057 (7)	0.0143 (7)	0.0016 (7)
N3B	0.0423 (8)	0.0590 (10)	0.0383 (8)	−0.0057 (7)	0.0129 (7)	−0.0020 (7)
N4B	0.0412 (8)	0.0561 (10)	0.0398 (8)	0.0019 (7)	0.0135 (7)	0.0012 (7)
C1B	0.0537 (13)	0.0992 (18)	0.0872 (16)	0.0014 (12)	0.0252 (12)	−0.0178 (13)
C2B	0.0789 (15)	0.0823 (15)	0.0762 (15)	−0.0032 (12)	0.0431 (12)	−0.0137 (12)
C3B	0.0504 (11)	0.0492 (11)	0.0664 (13)	−0.0030 (9)	0.0216 (10)	−0.0132 (10)
C4B	0.0500 (11)	0.0502 (11)	0.0535 (11)	−0.0012 (9)	0.0145 (9)	−0.0032 (9)
C5B	0.0477 (10)	0.0437 (10)	0.0482 (11)	−0.0008 (8)	0.0166 (9)	−0.0007 (8)
C6B	0.0522 (11)	0.0453 (10)	0.0475 (11)	−0.0007 (8)	0.0170 (9)	−0.0062 (8)
C7B	0.0673 (13)	0.0488 (11)	0.0470 (11)	−0.0003 (9)	0.0229 (10)	−0.0071 (8)
C8B	0.0611 (12)	0.0443 (11)	0.0622 (13)	−0.0059 (9)	0.0293 (10)	−0.0128 (9)
C9B	0.0479 (10)	0.0641 (12)	0.0482 (11)	0.0050 (9)	0.0132 (9)	0.0087 (9)
C10B	0.0584 (12)	0.0650 (13)	0.0406 (10)	0.0036 (10)	0.0139 (9)	−0.0066 (9)
C11B	0.0409 (10)	0.0682 (13)	0.0334 (9)	−0.0075 (10)	0.0153 (8)	0.0048 (9)
C12B	0.0392 (10)	0.0614 (12)	0.0352 (10)	−0.0035 (9)	0.0087 (8)	−0.0008 (9)
C13B	0.0380 (9)	0.0580 (11)	0.0381 (10)	−0.0041 (8)	0.0100 (8)	0.0013 (8)
C14B	0.0461 (11)	0.0640 (13)	0.0482 (12)	−0.0051 (9)	0.0088 (9)	0.0003 (10)
C15B	0.0674 (13)	0.0936 (17)	0.0682 (14)	−0.0333 (12)	0.0246 (11)	0.0037 (12)
C16B	0.112 (2)	0.0737 (17)	0.0967 (19)	−0.0279 (15)	0.0226 (16)	−0.0022 (14)
C17B	0.0378 (9)	0.0603 (11)	0.0389 (10)	−0.0037 (8)	0.0139 (8)	0.0000 (8)
C18B	0.0462 (11)	0.0665 (13)	0.0525 (12)	−0.0008 (10)	0.0215 (10)	0.0026 (10)
C19B	0.0712 (15)	0.150 (2)	0.0721 (16)	0.0005 (15)	0.0476 (13)	0.0227 (15)
C20B	0.134 (2)	0.109 (2)	0.0802 (18)	0.0127 (16)	0.0710 (17)	0.0114 (14)
C21B	0.0626 (12)	0.0641 (13)	0.0420 (10)	−0.0079 (10)	0.0202 (9)	−0.0094 (9)
C22B	0.0473 (11)	0.0665 (13)	0.0497 (11)	0.0079 (9)	0.0172 (9)	0.0000 (9)
C23B	0.0567 (11)	0.0522 (11)	0.0412 (10)	−0.0011 (9)	0.0211 (9)	−0.0074 (8)
C24B	0.0518 (11)	0.0520 (11)	0.0455 (11)	0.0042 (9)	0.0190 (9)	−0.0049 (9)
C25B	0.0654 (13)	0.0560 (12)	0.0503 (11)	0.0063 (10)	0.0173 (10)	0.0013 (9)
C26B	0.0751 (14)	0.0485 (12)	0.0605 (13)	−0.0005 (10)	0.0285 (11)	0.0000 (10)
C27B	0.0637 (12)	0.0450 (11)	0.0616 (13)	−0.0013 (9)	0.0249 (11)	−0.0067 (9)
C28B	0.0603 (12)	0.0511 (11)	0.0478 (11)	0.0000 (9)	0.0167 (9)	−0.0068 (9)
C29B	0.0784 (15)	0.0599 (14)	0.0920 (16)	−0.0143 (11)	0.0240 (13)	0.0021 (12)
C30B	0.1126 (19)	0.0788 (16)	0.0806 (16)	−0.0167 (14)	0.0272 (14)	0.0201 (13)

### Geometric parameters (Å, °)

**Table:**

O1A—C11A	1.2160 (18)	C27A—C28A	1.388 (3)
O2A—C12A	1.211 (2)	C27A—C29A	1.520 (3)
N1A—C11A	1.359 (2)	C28A—H28A	0.9300
N1A—C13A	1.4435 (19)	C29A—H29D	0.9600
N1A—C9A	1.452 (2)	C29A—H29E	0.9600
N2A—C12A	1.386 (2)	C29A—H29F	0.9600
N2A—C13A	1.432 (2)	C30A—H30D	0.9600
N2A—C10A	1.461 (2)	C30A—H30F	0.9600
N3A—C11A	1.382 (2)	C30A—H30E	0.9600
N3A—C17A	1.434 (2)	O1B—C11B	1.2143 (19)
N3A—C21A	1.466 (2)	O2B—C12B	1.2155 (19)
N4A—C12A	1.371 (2)	O3B—C14B	1.197 (2)
N4A—C17A	1.444 (2)	O4B—C14B	1.315 (2)
N4A—C22A	1.457 (2)	O4B—C15B	1.470 (2)
C1A—C3A	1.509 (2)	O5B—C18B	1.189 (2)
C1A—H1A1	0.9600	O6B—C18B	1.319 (2)
C1A—H1A2	0.9600	O6B—C19B	1.463 (2)
C1A—H1A3	0.9600	N1B—C11B	1.382 (2)
C2A—C8A	1.501 (2)	N1B—C13B	1.431 (2)
C2A—H2A1	0.9600	N1B—C9B	1.455 (2)
C2A—H2A2	0.9600	N2B—C12B	1.360 (2)
C2A—H2A3	0.9600	N2B—C13B	1.4434 (19)
C3A—C8A	1.389 (2)	N2B—C10B	1.457 (2)
C3A—C4A	1.390 (2)	N3B—C11B	1.365 (2)
C4A—C5A	1.383 (2)	N3B—C17B	1.443 (2)
C4A—H4A	0.9300	N3B—C21B	1.449 (2)
C5A—C6A	1.398 (2)	N4B—C12B	1.379 (2)
C5A—C9A	1.511 (2)	N4B—C17B	1.443 (2)
C6A—C7A	1.384 (2)	N4B—C22B	1.467 (2)
C6A—C10A	1.515 (2)	C1B—C3B	1.507 (3)
C7A—C8A	1.395 (2)	C1B—H1B1	0.9600
C7A—H7A	0.9300	C1B—H1B2	0.9600
C9A—H9A1	0.9700	C1B—H1B3	0.9600
C9A—H9A2	0.9700	C2B—C8B	1.512 (2)
C10A—H10C	0.9700	C2B—H2B1	0.9600
C10A—H10D	0.9700	C2B—H2B2	0.9600
C13A—C14A	1.543 (2)	C2B—H2B3	0.9600
C13A—C17A	1.570 (2)	C3B—C4B	1.380 (2)
C14A—O3'	1.200 (7)	C3B—C8B	1.394 (3)
C14A—O3A	1.213 (6)	C4B—C5B	1.388 (2)
C14A—O4A	1.291 (7)	C4B—H4B	0.9300
C14A—O4'	1.346 (7)	C5B—C6B	1.394 (2)
C15A—O4A	1.456 (7)	C5B—C9B	1.514 (2)
C15A—C16A	1.494 (8)	C6B—C7B	1.393 (2)
C15A—H15C	0.9700	C6B—C10B	1.511 (2)
C15A—H15D	0.9700	C7B—C8B	1.386 (2)
C16A—H16G	0.9600	C7B—H7B	0.9300
C16A—H16H	0.9600	C9B—H9B1	0.9700
C16A—H16I	0.9600	C9B—H9B2	0.9700
C16'—C15'	1.490 (8)	C10B—H10A	0.9700
C16'—H16F	0.9600	C10B—H10B	0.9700
C16'—H16E	0.9600	C13B—C14B	1.540 (2)
C16'—H16D	0.9600	C13B—C17B	1.563 (2)
C15'—O4'	1.461 (7)	C15B—C16B	1.488 (3)
C15'—H15E	0.9700	C15B—H15A	0.9700
C15'—H15F	0.9700	C15B—H15B	0.9700
C17A—C18A	1.540 (2)	C16B—H16A	0.9600
C18A—O5'	1.208 (5)	C16B—H16B	0.9600
C18A—O5A	1.216 (6)	C16B—H16C	0.9600
C18A—O6'	1.264 (5)	C17B—C18B	1.543 (2)
C18A—O6A	1.355 (8)	C19B—C20B	1.461 (3)
C19A—O6A	1.434 (8)	C19B—H19A	0.9700
C19A—C20A	1.500 (9)	C19B—H19B	0.9700
C19A—H19D	0.9700	C20B—H20G	0.9600
C19A—H19C	0.9700	C20B—H20H	0.9600
C20A—H20A	0.9600	C20B—H20I	0.9600
C20A—H20B	0.9600	C21B—C23B	1.508 (2)
C20A—H20C	0.9600	C21B—H21A	0.9700
C19'—O6'	1.455 (6)	C21B—H21B	0.9700
C19'—C20'	1.491 (7)	C22B—C24B	1.513 (2)
C19'—H19E	0.9700	C22B—H22A	0.9700
C19'—H19F	0.9700	C22B—H22B	0.9700
C20'—H20D	0.9600	C23B—C28B	1.388 (2)
C20'—H20E	0.9600	C23B—C24B	1.397 (2)
C20'—H20F	0.9600	C24B—C25B	1.380 (2)
C21A—C23A	1.509 (2)	C25B—C26B	1.391 (3)
C21A—H21C	0.9700	C25B—H25B	0.9300
C21A—H21D	0.9700	C26B—C27B	1.389 (3)
C22A—C24A	1.513 (2)	C26B—C30B	1.519 (3)
C22A—H22C	0.9700	C27B—C28B	1.385 (2)
C22A—H22D	0.9700	C27B—C29B	1.505 (3)
C23A—C28A	1.385 (2)	C28B—H28B	0.9300
C23A—C24A	1.394 (3)	C29B—H29A	0.9600
C24A—C25A	1.391 (2)	C29B—H29B	0.9600
C25A—C26A	1.389 (3)	C29B—H29C	0.9600
C25A—H25A	0.9300	C30B—H30A	0.9600
C26A—C27A	1.385 (3)	C30B—H30B	0.9600
C26A—C30A	1.512 (3)	C30B—H30C	0.9600
			
C11A—N1A—C13A	113.29 (14)	C27A—C29A—H29D	109.5
C11A—N1A—C9A	124.30 (14)	C27A—C29A—H29E	109.5
C13A—N1A—C9A	122.28 (13)	H29D—C29A—H29E	109.5
C12A—N2A—C13A	110.77 (14)	C27A—C29A—H29F	109.5
C12A—N2A—C10A	119.25 (15)	H29D—C29A—H29F	109.5
C13A—N2A—C10A	120.65 (14)	H29E—C29A—H29F	109.5
C11A—N3A—C17A	110.49 (14)	C26A—C30A—H30D	109.5
C11A—N3A—C21A	120.08 (14)	C26A—C30A—H30F	109.5
C17A—N3A—C21A	120.43 (14)	H30D—C30A—H30F	109.5
C12A—N4A—C17A	113.23 (14)	C26A—C30A—H30E	109.5
C12A—N4A—C22A	124.49 (15)	H30D—C30A—H30E	109.5
C17A—N4A—C22A	122.17 (14)	H30F—C30A—H30E	109.5
C3A—C1A—H1A1	109.5	C14B—O4B—C15B	116.61 (14)
C3A—C1A—H1A2	109.5	C18B—O6B—C19B	116.05 (15)
H1A1—C1A—H1A2	109.5	C11B—N1B—C13B	110.60 (14)
C3A—C1A—H1A3	109.5	C11B—N1B—C9B	121.18 (14)
H1A1—C1A—H1A3	109.5	C13B—N1B—C9B	120.54 (14)
H1A2—C1A—H1A3	109.5	C12B—N2B—C13B	112.36 (13)
C8A—C2A—H2A1	109.5	C12B—N2B—C10B	124.30 (14)
C8A—C2A—H2A2	109.5	C13B—N2B—C10B	122.21 (14)
H2A1—C2A—H2A2	109.5	C11B—N3B—C17B	112.89 (14)
C8A—C2A—H2A3	109.5	C11B—N3B—C21B	124.17 (15)
H2A1—C2A—H2A3	109.5	C17B—N3B—C21B	122.92 (14)
H2A2—C2A—H2A3	109.5	C12B—N4B—C17B	110.61 (14)
C8A—C3A—C4A	118.43 (15)	C12B—N4B—C22B	119.41 (14)
C8A—C3A—C1A	122.93 (16)	C17B—N4B—C22B	120.20 (13)
C4A—C3A—C1A	118.65 (16)	C3B—C1B—H1B1	109.5
C5A—C4A—C3A	123.22 (16)	C3B—C1B—H1B2	109.5
C5A—C4A—H4A	118.4	H1B1—C1B—H1B2	109.5
C3A—C4A—H4A	118.4	C3B—C1B—H1B3	109.5
C4A—C5A—C6A	118.58 (15)	H1B1—C1B—H1B3	109.5
C4A—C5A—C9A	119.55 (15)	H1B2—C1B—H1B3	109.5
C6A—C5A—C9A	121.85 (14)	C8B—C2B—H2B1	109.5
C7A—C6A—C5A	118.13 (15)	C8B—C2B—H2B2	109.5
C7A—C6A—C10A	119.43 (15)	H2B1—C2B—H2B2	109.5
C5A—C6A—C10A	122.39 (15)	C8B—C2B—H2B3	109.5
C6A—C7A—C8A	123.30 (16)	H2B1—C2B—H2B3	109.5
C6A—C7A—H7A	118.3	H2B2—C2B—H2B3	109.5
C8A—C7A—H7A	118.3	C4B—C3B—C8B	118.02 (17)
C3A—C8A—C7A	118.31 (16)	C4B—C3B—C1B	120.04 (18)
C3A—C8A—C2A	122.26 (16)	C8B—C3B—C1B	121.92 (17)
C7A—C8A—C2A	119.43 (16)	C3B—C4B—C5B	123.67 (17)
N1A—C9A—C5A	113.18 (13)	C3B—C4B—H4B	118.2
N1A—C9A—H9A1	108.9	C5B—C4B—H4B	118.2
C5A—C9A—H9A1	108.9	C4B—C5B—C6B	118.33 (16)
N1A—C9A—H9A2	108.9	C4B—C5B—C9B	117.57 (15)
C5A—C9A—H9A2	108.9	C6B—C5B—C9B	123.85 (15)
H9A1—C9A—H9A2	107.8	C7B—C6B—C5B	118.06 (16)
N2A—C10A—C6A	115.70 (13)	C7B—C6B—C10B	117.99 (16)
N2A—C10A—H10C	108.4	C5B—C6B—C10B	123.84 (15)
C6A—C10A—H10C	108.4	C8B—C7B—C6B	123.10 (17)
N2A—C10A—H10D	108.4	C8B—C7B—H7B	118.5
C6A—C10A—H10D	108.4	C6B—C7B—H7B	118.5
H10C—C10A—H10D	107.4	C7B—C8B—C3B	118.72 (17)
O1A—C11A—N1A	126.20 (16)	C7B—C8B—C2B	119.80 (18)
O1A—C11A—N3A	125.68 (16)	C3B—C8B—C2B	121.48 (18)
N1A—C11A—N3A	108.10 (14)	N1B—C9B—C5B	117.44 (14)
O2A—C12A—N4A	126.46 (18)	N1B—C9B—H9B1	107.9
O2A—C12A—N2A	125.99 (18)	C5B—C9B—H9B1	107.9
N4A—C12A—N2A	107.54 (16)	N1B—C9B—H9B2	107.9
N2A—C13A—N1A	114.10 (13)	C5B—C9B—H9B2	107.9
N2A—C13A—C14A	114.70 (15)	H9B1—C9B—H9B2	107.2
N1A—C13A—C14A	108.67 (14)	N2B—C10B—C6B	115.36 (14)
N2A—C13A—C17A	103.51 (13)	N2B—C10B—H10A	108.4
N1A—C13A—C17A	100.93 (12)	C6B—C10B—H10A	108.4
C14A—C13A—C17A	114.13 (14)	N2B—C10B—H10B	108.4
O3'—C14A—O4A	114.8 (7)	C6B—C10B—H10B	108.4
O3A—C14A—O4A	122.9 (7)	H10A—C10B—H10B	107.5
O3'—C14A—O4'	127.2 (7)	O1B—C11B—N3B	126.51 (18)
O3A—C14A—O4'	125.9 (8)	O1B—C11B—N1B	125.81 (18)
O3'—C14A—C13A	123.0 (6)	N3B—C11B—N1B	107.65 (15)
O3A—C14A—C13A	120.1 (5)	O2B—C12B—N2B	126.48 (16)
O4A—C14A—C13A	117.0 (5)	O2B—C12B—N4B	125.03 (17)
O4'—C14A—C13A	109.3 (5)	N2B—C12B—N4B	108.48 (14)
O4A—C15A—C16A	106.7 (7)	N1B—C13B—N2B	113.60 (13)
O4A—C15A—H15C	110.4	N1B—C13B—C14B	113.69 (14)
C16A—C15A—H15C	110.4	N2B—C13B—C14B	110.33 (14)
O4A—C15A—H15D	110.4	N1B—C13B—C17B	103.02 (13)
C16A—C15A—H15D	110.4	N2B—C13B—C17B	101.48 (13)
H15C—C15A—H15D	108.6	C14B—C13B—C17B	113.95 (13)
C14A—O4A—C15A	114.5 (8)	O3B—C14B—O4B	126.18 (17)
C15'—C16'—H16F	109.5	O3B—C14B—C13B	121.69 (17)
C15'—C16'—H16E	109.5	O4B—C14B—C13B	112.13 (15)
H16F—C16'—H16E	109.5	O4B—C15B—C16B	111.87 (17)
C15'—C16'—H16D	109.5	O4B—C15B—H15A	109.2
H16F—C16'—H16D	109.5	C16B—C15B—H15A	109.2
H16E—C16'—H16D	109.5	O4B—C15B—H15B	109.2
O4'—C15'—C16'	111.8 (7)	C16B—C15B—H15B	109.2
O4'—C15'—H15E	109.3	H15A—C15B—H15B	107.9
C16'—C15'—H15E	109.3	C15B—C16B—H16A	109.5
O4'—C15'—H15F	109.3	C15B—C16B—H16B	109.5
C16'—C15'—H15F	109.3	H16A—C16B—H16B	109.5
H15E—C15'—H15F	107.9	C15B—C16B—H16C	109.5
C14A—O4'—C15'	119.3 (8)	H16A—C16B—H16C	109.5
N3A—C17A—N4A	113.99 (13)	H16B—C16B—H16C	109.5
N3A—C17A—C18A	111.39 (15)	N3B—C17B—N4B	114.56 (13)
N4A—C17A—C18A	112.37 (15)	N3B—C17B—C18B	113.43 (14)
N3A—C17A—C13A	103.45 (13)	N4B—C17B—C18B	110.67 (14)
N4A—C17A—C13A	101.09 (13)	N3B—C17B—C13B	101.25 (12)
C18A—C17A—C13A	113.87 (15)	N4B—C17B—C13B	102.69 (13)
O5'—C18A—O6'	125.4 (5)	C18B—C17B—C13B	113.46 (14)
O5A—C18A—O6'	114.8 (6)	O5B—C18B—O6B	125.66 (16)
O5'—C18A—O6A	119.9 (7)	O5B—C18B—C17B	122.84 (17)
O5A—C18A—O6A	125.8 (7)	O6B—C18B—C17B	111.46 (16)
O5'—C18A—C17A	122.2 (4)	C20B—C19B—O6B	108.48 (18)
O5A—C18A—C17A	122.6 (5)	C20B—C19B—H19A	110.0
O6'—C18A—C17A	112.0 (4)	O6B—C19B—H19A	110.0
O6A—C18A—C17A	110.0 (5)	C20B—C19B—H19B	110.0
O6A—C19A—C20A	110.2 (8)	O6B—C19B—H19B	110.0
O6A—C19A—H19D	109.6	H19A—C19B—H19B	108.4
C20A—C19A—H19D	109.6	C19B—C20B—H20G	109.5
O6A—C19A—H19C	109.6	C19B—C20B—H20H	109.5
C20A—C19A—H19C	109.6	H20G—C20B—H20H	109.5
H19D—C19A—H19C	108.1	C19B—C20B—H20I	109.5
C18A—O6A—C19A	122.3 (10)	H20G—C20B—H20I	109.5
O6'—C19'—C20'	106.8 (6)	H20H—C20B—H20I	109.5
O6'—C19'—H19E	110.4	N3B—C21B—C23B	112.75 (14)
C20'—C19'—H19E	110.4	N3B—C21B—H21A	109.0
O6'—C19'—H19F	110.4	C23B—C21B—H21A	109.0
C20'—C19'—H19F	110.4	N3B—C21B—H21B	109.0
H19E—C19'—H19F	108.6	C23B—C21B—H21B	109.0
C19'—C20'—H20D	109.5	H21A—C21B—H21B	107.8
C19'—C20'—H20E	109.5	N4B—C22B—C24B	113.86 (14)
H20D—C20'—H20E	109.5	N4B—C22B—H22A	108.8
C19'—C20'—H20F	109.5	C24B—C22B—H22A	108.8
H20D—C20'—H20F	109.5	N4B—C22B—H22B	108.8
H20E—C20'—H20F	109.5	C24B—C22B—H22B	108.8
C18A—O6'—C19'	117.4 (6)	H22A—C22B—H22B	107.7
N3A—C21A—C23A	115.69 (15)	C28B—C23B—C24B	118.74 (16)
N3A—C21A—H21C	108.4	C28B—C23B—C21B	120.28 (16)
C23A—C21A—H21C	108.4	C24B—C23B—C21B	120.97 (16)
N3A—C21A—H21D	108.4	C25B—C24B—C23B	118.46 (17)
C23A—C21A—H21D	108.4	C25B—C24B—C22B	120.00 (16)
H21C—C21A—H21D	107.4	C23B—C24B—C22B	121.53 (16)
N4A—C22A—C24A	113.69 (14)	C24B—C25B—C26B	122.67 (18)
N4A—C22A—H22C	108.8	C24B—C25B—H25B	118.7
C24A—C22A—H22C	108.8	C26B—C25B—H25B	118.7
N4A—C22A—H22D	108.8	C27B—C26B—C25B	118.94 (17)
C24A—C22A—H22D	108.8	C27B—C26B—C30B	121.43 (19)
H22C—C22A—H22D	107.7	C25B—C26B—C30B	119.61 (19)
C28A—C23A—C24A	118.49 (18)	C28B—C27B—C26B	118.48 (17)
C28A—C23A—C21A	118.96 (18)	C28B—C27B—C29B	119.65 (18)
C24A—C23A—C21A	122.52 (17)	C26B—C27B—C29B	121.84 (18)
C25A—C24A—C23A	118.19 (18)	C27B—C28B—C23B	122.69 (17)
C25A—C24A—C22A	119.97 (18)	C27B—C28B—H28B	118.7
C23A—C24A—C22A	121.84 (17)	C23B—C28B—H28B	118.7
C26A—C25A—C24A	123.2 (2)	C27B—C29B—H29A	109.5
C26A—C25A—H25A	118.4	C27B—C29B—H29B	109.5
C24A—C25A—H25A	118.4	H29A—C29B—H29B	109.5
C27A—C26A—C25A	118.32 (19)	C27B—C29B—H29C	109.5
C27A—C26A—C30A	122.2 (2)	H29A—C29B—H29C	109.5
C25A—C26A—C30A	119.4 (2)	H29B—C29B—H29C	109.5
C26A—C27A—C28A	118.79 (19)	C26B—C30B—H30A	109.5
C26A—C27A—C29A	121.4 (2)	C26B—C30B—H30B	109.5
C28A—C27A—C29A	119.7 (2)	H30A—C30B—H30B	109.5
C23A—C28A—C27A	123.0 (2)	C26B—C30B—H30C	109.5
C23A—C28A—H28A	118.5	H30A—C30B—H30C	109.5
C27A—C28A—H28A	118.5	H30B—C30B—H30C	109.5
			
C8A—C3A—C4A—C5A	1.3 (3)	N4A—C22A—C24A—C23A	−53.5 (2)
C1A—C3A—C4A—C5A	−179.06 (16)	C23A—C24A—C25A—C26A	1.5 (3)
C3A—C4A—C5A—C6A	0.1 (2)	C22A—C24A—C25A—C26A	−178.75 (16)
C3A—C4A—C5A—C9A	178.31 (15)	C24A—C25A—C26A—C27A	0.4 (3)
C4A—C5A—C6A—C7A	−1.5 (2)	C24A—C25A—C26A—C30A	179.80 (17)
C9A—C5A—C6A—C7A	−179.68 (15)	C25A—C26A—C27A—C28A	−1.4 (3)
C4A—C5A—C6A—C10A	175.81 (15)	C30A—C26A—C27A—C28A	179.23 (18)
C9A—C5A—C6A—C10A	−2.3 (2)	C25A—C26A—C27A—C29A	176.23 (18)
C5A—C6A—C7A—C8A	1.7 (2)	C30A—C26A—C27A—C29A	−3.1 (3)
C10A—C6A—C7A—C8A	−175.76 (15)	C24A—C23A—C28A—C27A	1.4 (3)
C4A—C3A—C8A—C7A	−1.2 (2)	C21A—C23A—C28A—C27A	−176.58 (17)
C1A—C3A—C8A—C7A	179.18 (16)	C26A—C27A—C28A—C23A	0.5 (3)
C4A—C3A—C8A—C2A	179.21 (16)	C29A—C27A—C28A—C23A	−177.18 (19)
C1A—C3A—C8A—C2A	−0.5 (3)	C8B—C3B—C4B—C5B	2.6 (3)
C6A—C7A—C8A—C3A	−0.3 (3)	C1B—C3B—C4B—C5B	−176.01 (17)
C6A—C7A—C8A—C2A	179.36 (16)	C3B—C4B—C5B—C6B	−2.7 (3)
C11A—N1A—C9A—C5A	−106.97 (17)	C3B—C4B—C5B—C9B	171.81 (16)
C13A—N1A—C9A—C5A	77.30 (18)	C4B—C5B—C6B—C7B	0.2 (2)
C4A—C5A—C9A—N1A	126.93 (16)	C9B—C5B—C6B—C7B	−173.89 (16)
C6A—C5A—C9A—N1A	−54.9 (2)	C4B—C5B—C6B—C10B	176.46 (16)
C12A—N2A—C10A—C6A	71.0 (2)	C9B—C5B—C6B—C10B	2.3 (3)
C13A—N2A—C10A—C6A	−72.5 (2)	C5B—C6B—C7B—C8B	2.3 (3)
C7A—C6A—C10A—N2A	−124.46 (17)	C10B—C6B—C7B—C8B	−174.17 (16)
C5A—C6A—C10A—N2A	58.2 (2)	C6B—C7B—C8B—C3B	−2.4 (3)
C13A—N1A—C11A—O1A	178.27 (16)	C6B—C7B—C8B—C2B	177.66 (16)
C9A—N1A—C11A—O1A	2.2 (3)	C4B—C3B—C8B—C7B	0.0 (3)
C13A—N1A—C11A—N3A	−0.24 (18)	C1B—C3B—C8B—C7B	178.54 (17)
C9A—N1A—C11A—N3A	−176.31 (14)	C4B—C3B—C8B—C2B	179.91 (16)
C17A—N3A—C11A—O1A	168.77 (16)	C1B—C3B—C8B—C2B	−1.5 (3)
C21A—N3A—C11A—O1A	21.5 (3)	C11B—N1B—C9B—C5B	−76.6 (2)
C17A—N3A—C11A—N1A	−12.71 (18)	C13B—N1B—C9B—C5B	70.0 (2)
C21A—N3A—C11A—N1A	−159.99 (14)	C4B—C5B—C9B—N1B	134.07 (16)
C17A—N4A—C12A—O2A	179.59 (16)	C6B—C5B—C9B—N1B	−51.8 (2)
C22A—N4A—C12A—O2A	3.3 (3)	C12B—N2B—C10B—C6B	120.76 (17)
C17A—N4A—C12A—N2A	0.43 (18)	C13B—N2B—C10B—C6B	−72.4 (2)
C22A—N4A—C12A—N2A	−175.90 (14)	C7B—C6B—C10B—N2B	−135.78 (16)
C13A—N2A—C12A—O2A	167.42 (16)	C5B—C6B—C10B—N2B	48.0 (2)
C10A—N2A—C12A—O2A	20.6 (2)	C17B—N3B—C11B—O1B	−179.01 (15)
C13A—N2A—C12A—N4A	−13.43 (17)	C21B—N3B—C11B—O1B	2.8 (3)
C10A—N2A—C12A—N4A	−160.29 (13)	C17B—N3B—C11B—N1B	−0.94 (17)
C12A—N2A—C13A—N1A	−89.10 (16)	C21B—N3B—C11B—N1B	−179.15 (13)
C10A—N2A—C13A—N1A	57.23 (19)	C13B—N1B—C11B—O1B	−166.92 (15)
C12A—N2A—C13A—C14A	144.62 (15)	C9B—N1B—C11B—O1B	−17.4 (2)
C10A—N2A—C13A—C14A	−69.05 (19)	C13B—N1B—C11B—N3B	14.99 (17)
C12A—N2A—C13A—C17A	19.63 (16)	C9B—N1B—C11B—N3B	164.52 (13)
C10A—N2A—C13A—C17A	165.96 (13)	C13B—N2B—C12B—O2B	−174.47 (16)
C11A—N1A—C13A—N2A	121.71 (15)	C10B—N2B—C12B—O2B	−6.5 (3)
C9A—N1A—C13A—N2A	−62.13 (19)	C13B—N2B—C12B—N4B	5.16 (18)
C11A—N1A—C13A—C14A	−108.92 (16)	C10B—N2B—C12B—N4B	173.16 (14)
C9A—N1A—C13A—C14A	67.24 (19)	C17B—N4B—C12B—O2B	−170.80 (16)
C11A—N1A—C13A—C17A	11.40 (17)	C22B—N4B—C12B—O2B	−24.8 (2)
C9A—N1A—C13A—C17A	−172.44 (14)	C17B—N4B—C12B—N2B	9.56 (18)
N2A—C13A—C14A—O3'	177.6 (8)	C22B—N4B—C12B—N2B	155.54 (14)
N1A—C13A—C14A—O3'	48.6 (9)	C11B—N1B—C13B—N2B	87.41 (16)
C17A—C13A—C14A—O3'	−63.2 (9)	C9B—N1B—C13B—N2B	−62.35 (19)
N2A—C13A—C14A—O3A	147.2 (6)	C11B—N1B—C13B—C14B	−145.31 (14)
N1A—C13A—C14A—O3A	18.2 (6)	C9B—N1B—C13B—C14B	64.94 (19)
C17A—C13A—C14A—O3A	−93.6 (6)	C11B—N1B—C13B—C17B	−21.49 (16)
N2A—C13A—C14A—O4A	−29.2 (7)	C9B—N1B—C13B—C17B	−171.25 (13)
N1A—C13A—C14A—O4A	−158.3 (6)	C12B—N2B—C13B—N1B	−125.86 (15)
C17A—C13A—C14A—O4A	90.0 (7)	C10B—N2B—C13B—N1B	65.85 (19)
N2A—C13A—C14A—O4'	−9.8 (6)	C12B—N2B—C13B—C14B	105.12 (16)
N1A—C13A—C14A—O4'	−138.9 (6)	C10B—N2B—C13B—C14B	−63.17 (19)
C17A—C13A—C14A—O4'	109.4 (6)	C12B—N2B—C13B—C17B	−16.01 (17)
O3'—C14A—O4A—C15A	−23.4 (12)	C10B—N2B—C13B—C17B	175.70 (13)
O3A—C14A—O4A—C15A	4.9 (12)	C15B—O4B—C14B—O3B	0.4 (3)
O4'—C14A—O4A—C15A	110 (3)	C15B—O4B—C14B—C13B	179.78 (15)
C13A—C14A—O4A—C15A	−178.7 (6)	N1B—C13B—C14B—O3B	−149.65 (17)
C16A—C15A—O4A—C14A	−173.2 (15)	N2B—C13B—C14B—O3B	−20.7 (2)
O3'—C14A—O4'—C15'	−14.9 (14)	C17B—C13B—C14B—O3B	92.7 (2)
O3A—C14A—O4'—C15'	17.5 (12)	N1B—C13B—C14B—O4B	30.9 (2)
O4A—C14A—O4'—C15'	−70 (2)	N2B—C13B—C14B—O4B	159.89 (14)
C13A—C14A—O4'—C15'	172.9 (6)	C17B—C13B—C14B—O4B	−86.73 (18)
C16'—C15'—O4'—C14A	97.1 (17)	C14B—O4B—C15B—C16B	78.0 (2)
C11A—N3A—C17A—N4A	−89.73 (17)	C11B—N3B—C17B—N4B	−121.39 (15)
C21A—N3A—C17A—N4A	57.41 (19)	C21B—N3B—C17B—N4B	56.85 (19)
C11A—N3A—C17A—C18A	141.83 (15)	C11B—N3B—C17B—C18B	110.21 (16)
C21A—N3A—C17A—C18A	−71.0 (2)	C21B—N3B—C17B—C18B	−71.54 (19)
C11A—N3A—C17A—C13A	19.10 (17)	C11B—N3B—C17B—C13B	−11.69 (16)
C21A—N3A—C17A—C13A	166.24 (14)	C21B—N3B—C17B—C13B	166.55 (13)
C12A—N4A—C17A—N3A	121.29 (15)	C12B—N4B—C17B—N3B	90.12 (17)
C22A—N4A—C17A—N3A	−62.3 (2)	C22B—N4B—C17B—N3B	−55.55 (19)
C12A—N4A—C17A—C18A	−110.77 (17)	C12B—N4B—C17B—C18B	−140.11 (14)
C22A—N4A—C17A—C18A	65.7 (2)	C22B—N4B—C17B—C18B	74.22 (18)
C12A—N4A—C17A—C13A	11.01 (16)	C12B—N4B—C17B—C13B	−18.71 (16)
C22A—N4A—C17A—C13A	−172.56 (14)	C22B—N4B—C17B—C13B	−164.38 (13)
N2A—C13A—C17A—N3A	−135.92 (13)	N1B—C13B—C17B—N3B	19.17 (15)
N1A—C13A—C17A—N3A	−17.62 (15)	N2B—C13B—C17B—N3B	−98.63 (13)
C14A—C13A—C17A—N3A	98.73 (16)	C14B—C13B—C17B—N3B	142.81 (14)
N2A—C13A—C17A—N4A	−17.70 (15)	N1B—C13B—C17B—N4B	137.80 (12)
N1A—C13A—C17A—N4A	100.60 (13)	N2B—C13B—C17B—N4B	20.01 (14)
C14A—C13A—C17A—N4A	−143.05 (15)	C14B—C13B—C17B—N4B	−98.55 (15)
N2A—C13A—C17A—C18A	103.02 (16)	N1B—C13B—C17B—C18B	−102.72 (15)
N1A—C13A—C17A—C18A	−138.68 (15)	N2B—C13B—C17B—C18B	139.49 (14)
C14A—C13A—C17A—C18A	−22.3 (2)	C14B—C13B—C17B—C18B	20.9 (2)
N3A—C17A—C18A—O5'	−0.4 (8)	C19B—O6B—C18B—O5B	−0.4 (3)
N4A—C17A—C18A—O5'	−129.7 (8)	C19B—O6B—C18B—C17B	−178.18 (17)
C13A—C17A—C18A—O5'	116.2 (8)	N3B—C17B—C18B—O5B	165.61 (18)
N3A—C17A—C18A—O5A	−45.0 (8)	N4B—C17B—C18B—O5B	35.2 (2)
N4A—C17A—C18A—O5A	−174.3 (7)	C13B—C17B—C18B—O5B	−79.6 (2)
C13A—C17A—C18A—O5A	71.5 (7)	N3B—C17B—C18B—O6B	−16.5 (2)
N3A—C17A—C18A—O6'	172.4 (5)	N4B—C17B—C18B—O6B	−146.88 (15)
N4A—C17A—C18A—O6'	43.1 (5)	C13B—C17B—C18B—O6B	98.31 (17)
C13A—C17A—C18A—O6'	−71.1 (5)	C18B—O6B—C19B—C20B	−171.94 (18)
N3A—C17A—C18A—O6A	148.5 (6)	C11B—N3B—C21B—C23B	101.56 (18)
N4A—C17A—C18A—O6A	19.2 (6)	C17B—N3B—C21B—C23B	−76.48 (19)
C13A—C17A—C18A—O6A	−95.0 (6)	C12B—N4B—C22B—C24B	−66.68 (19)
O5'—C18A—O6A—C19A	−33.7 (11)	C17B—N4B—C22B—C24B	76.02 (18)
O5A—C18A—O6A—C19A	10.6 (12)	N3B—C21B—C23B—C28B	−118.73 (17)
O6'—C18A—O6A—C19A	77 (2)	N3B—C21B—C23B—C24B	60.3 (2)
C17A—C18A—O6A—C19A	176.6 (7)	C28B—C23B—C24B—C25B	−0.9 (2)
C20A—C19A—O6A—C18A	−97 (2)	C21B—C23B—C24B—C25B	−179.94 (16)
O5'—C18A—O6'—C19'	−9.7 (9)	C28B—C23B—C24B—C22B	177.98 (15)
O5A—C18A—O6'—C19'	32.2 (8)	C21B—C23B—C24B—C22B	−1.0 (2)
O6A—C18A—O6'—C19'	−93 (2)	N4B—C22B—C24B—C25B	118.61 (18)
C17A—C18A—O6'—C19'	177.9 (5)	N4B—C22B—C24B—C23B	−60.3 (2)
C20'—C19'—O6'—C18A	−178.2 (12)	C23B—C24B—C25B—C26B	0.3 (3)
C11A—N3A—C21A—C23A	71.3 (2)	C22B—C24B—C25B—C26B	−178.62 (16)
C17A—N3A—C21A—C23A	−72.7 (2)	C24B—C25B—C26B—C27B	0.7 (3)
C12A—N4A—C22A—C24A	−107.15 (19)	C24B—C25B—C26B—C30B	179.62 (18)
C17A—N4A—C22A—C24A	76.8 (2)	C25B—C26B—C27B—C28B	−1.1 (3)
N3A—C21A—C23A—C28A	−122.71 (18)	C30B—C26B—C27B—C28B	−179.96 (18)
N3A—C21A—C23A—C24A	59.4 (2)	C25B—C26B—C27B—C29B	177.13 (17)
C28A—C23A—C24A—C25A	−2.4 (3)	C30B—C26B—C27B—C29B	−1.7 (3)
C21A—C23A—C24A—C25A	175.58 (16)	C26B—C27B—C28B—C23B	0.5 (3)
C28A—C23A—C24A—C22A	177.89 (16)	C29B—C27B—C28B—C23B	−177.79 (16)
C21A—C23A—C24A—C22A	−4.2 (3)	C24B—C23B—C28B—C27B	0.6 (3)
N4A—C22A—C24A—C25A	126.78 (18)	C21B—C23B—C28B—C27B	179.57 (16)

### Hydrogen-bond geometry (Å, °)

**Table:**

*D*—H···*A*	*D*—H	H···*A*	*D*···*A*	*D*—H···*A*
C9A—H9A1···O2B^i^	0.97	2.31	3.205 (2)	153
C22B—H22A···O1A^ii^	0.97	2.41	3.375 (2)	178
C29B—H29A···O1B^iii^	0.96	2.53	3.441 (3)	160

Symmetry codes: (i) *x*, *y*+1, *z*; (ii) *x*, −*y*+3/2, *z*+1/2; (iii) −*x*+1, *y*+1/2, −*z*+3/2.
